# Evaluating the Alcohol Environment: Community Geography and Alcohol Problems

**Published:** 2002

**Authors:** Paul J. Gruenewald, Lillian Remer, Rob Lipton

**Affiliations:** Paul J. Gruenewald, Ph.D., is a senior research scientist and associate director; Lillian Remer, M.A., is a research associate; and Rob Lipton, Ph.D., is an associate research scientist, at the Prevention Research Center of the Pacific Institute for Research and Evaluation, Berkeley, California

In recent years, a growing number of studies in the United States have addressed the relationships among the environments in which people live, the alcoholic beverages they consume, and the problems they experience in different community settings. Such research arises from a view of community settings and alcohol problems that takes into account both individual drinking behaviors and the environmental contexts in which these behaviors occur. According to this approach, drinking in different settings (e.g., drinking at restaurants) exposes drinkers to different risks (e.g., driving after drinking), and these risks become greater with the continued use of alcohol (e.g., heavy drinking leading to driving while intoxicated). The availability of alcohol at different places where people may drink affects drinking practices and shapes the incidence, prevalence, and geographic distribution of alcohol-related problems in the community (Stockwell and Gruenewald 2001). The different places where drinkers may use alcohol also change in response to the demand for alcohol and in response to changes in community systems that meet this demand (i.e., changes in alcohol availability policy) (Holder 1998). Regulations and policies related to the availability and use of alcohol provide an opportunity for policymakers to affect the geographic distribution of alcohol problems and create safer communities (Gruenewald et al. in press).

Policymakers have had the opportunity to regulate the distribution of alcohol for the benefit of public health for many decades (Edwards et al. 1994). Research to evaluate the effectiveness of these efforts, however, has been limited by inadequate models of the individual-environmental interactions that support alcohol use and by imprecise views of how these interactions are related to the geographic distribution of alcohol use and related problems. Knowledge of where alcohol problems occur and why they occur in the places that they do is essential to local policymakers. Such knowledge can inform decisions as to what to regulate (e.g., alcohol beverage serving practices vs. restrictions on places that can serve alcohol) and where such regulation is most called for (e.g., bars or restaurants in downtown or outlying areas). This knowledge is also useful for researchers to consider when planning and evaluating community prevention programs. In recent years, studies of the geographic relationships between places where alcohol is sold and local alcohol-related problems have begun to flourish. These advances have been coupled with the development of computerized systems for storing and mapping data known as geographic information systems (GIS) and geostatistical methods (Wilson and Dufour 2000). Although still at its earliest stages, geographical analysis has begun to reveal very useful spatial associations between features of the alcohol environment and problems related to alcohol, particularly motor vehicle crashes, pedestrian injuries, and violence.

Most current ecological studies of the interactions of individual drinking practices with the drinking environment are rooted in the simple observation that alcohol problems occur in environmental settings, and environmental settings may be changed through community action. The question these studies attempt to answer, then, is this: What specific changes in the alcohol environment should be recommended to communities? Some preliminary answers to this question are available, and several of them are discussed in this issue of *Alcohol Research & Health.* A more complete answer to this question, however, will require considerably more work. The full effects of environmental changes in community settings are not well understood. Current research provides researchers and policymakers with early demonstrations of relationships between environmental settings and drinking practices. This work has not, however, clarified the specific components of these ecological relationships that are most productive of alcohol-related problems. For example, there is evidence that decreased outlet densities are related to decreased sales and fewer problem outcomes. But are reductions in outlet densities always related to reductions in problems? The answer may be “no” and may be contingent on other local environmental conditions (e.g., in the case of crashes, traffic flow around outlets may be an important local environmental condition) (Gruenewald and Treno 2000). Fortunately, technological improvements in computer-based GIS, increased knowledge about statistical procedures for handling spatial data, and a developing understanding of the ecologies of alcohol-related problems now make it possible to pursue these important issues in the environmental regulation of alcohol use and related problems.

This sidebar examines the research techniques used to study the geography of alcohol use and related problems in a community and describes ways that these techniques may be used to prevent alcohol-related problems.

## Understanding Drinking Environments

There are three complementary ways of obtaining information about the role of the alcohol environment in alcohol use and related problems: asking people when and where they drink, examining the geographic distribution of drinking-related events, and obtaining information on drinking places and their relationships to drinking outcomes. The first and most customary way to gather information about drinking environments is simply to ask people where they drink, the circumstances of their drinking, and their drinking behaviors. This survey approach collects general information about the use of drinking venues (e.g., bars, restaurants, at home), the relationships between drinking patterns and drinking locations, and the relationships between drinking behaviors and problem outcomes (e.g., Single and Wortley 1993). Surveys are useful for studying how people interact with their environments, such as whether people purchase alcohol along with other goods or whether beer drinkers are more likely than wine drinkers to drink and drive.

The second way of obtaining information about drinking environments is to examine the geographic distribution of drinking-related events, such as the locations of alcohol-related crashes, arrests for public drunkenness, and other alcohol-related problems. This approach draws on archival data, such as police reports, and emphasizes the importance of mapping to understanding alcohol problems. If there is a regular and predictable geographic distribution of problem events (e.g., alcohol-related crashes), then some feature of the environment (e.g., locations of high traffic flow) must be related to those problems.

The third approach to obtaining information about the alcohol environment and its relationships to alcohol problems is more difficult. It requires the identification of specific environmental features of the community related to alcohol use (e.g., alcohol outlets), and the empirical examination of the relationships between these environmental features and problem outcomes. This third approach is particularly useful in cases where neighborhood alcohol problems are not necessarily related to the drinking behavior of local residents (as typically assessed using survey techniques). Alcohol-related motor vehicle crashes, for instance, may be more prevalent in areas near alcohol outlets but may be attributable to the drinking behaviors of nonresidents who frequent local establishments (Gruenewald et al. 1996). Thus, survey data on drinking and driving in a given geographic area may have no bearing on local rates of alcohol crashes but may bear upon crashes someplace else. Complementarily, high rates of crashes in some neighborhoods may be attributed to environmental features located elsewhere. Thus, neighborhoods adjacent to areas of high outlet density have high alcohol-related crash rates (Gruenewald et al. 1996). Analyses of geographic data may therefore reveal associations between the use of alcohol outlets and problem outcomes, such as violence, in which local residents become the victims of problems related to the use of alcohol by someone else (Gorman et al. 2001) or to the availability of alcohol someplace else (e.g., alcohol-related crashes) (Gruenewald et al. 1996). Only the combined use of survey and archival data on problem events and drinking places will begin to reveal these interrelationships in a spatial ecology of community alcohol problems. The tools and techniques described below provide the methods necessary to this approach.

## Maps and Mapping

**Figure 1:**
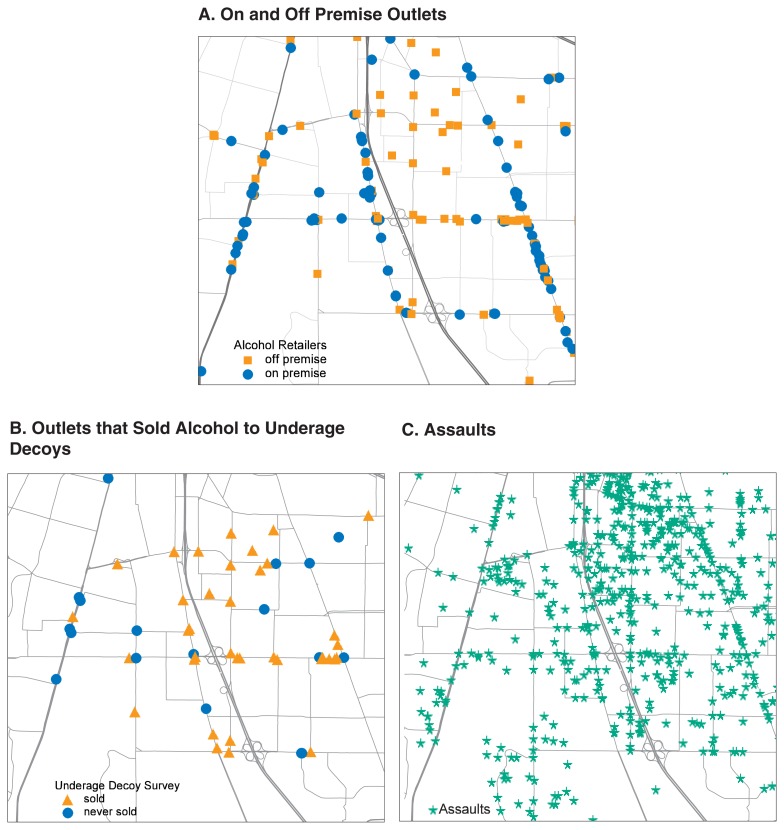
Illustration of the use of maps and mapping in alcohol research. These tools can be used to study the locations of alcohol outlets and alcohol-related problems. Figure 1a shows all the alcohol outlets in a given geographic area. Outlets that sold alcohol to underage decoys are shown in figure 1b. As shown in figure 1c, assaults tend to cluster around these outlet locations. NOTE: Figure 1a–On premise outlets (bars) serve alcoholic beverages. Off premise outlets (stores) sell alcoholic beverages for consumption elsewhere. Figure 1b–Underage decoys are people under age 21 who were working with the police.

Maps and mapping are used in alcohol research to study the locations of alcohol outlets and the locations of alcohol-related problems. These environmental objects and events are distributed across communities in predictable ways and can be mapped using GIS. For example, figure 1a shows all the alcohol outlets in a given geographic area. Outlets that sold alcohol to underage decoys—people under age 21 working with the police—are shown in figure 1b. As shown in figure 1c, assaults tend to cluster around these outlet locations. Maps tell police where crimes may be more likely to occur, inform researchers where to start looking for the cause of alcohol-related problems, and guide policymakers concerning interventions.

## Spatial Analysis

**Figure 2:**
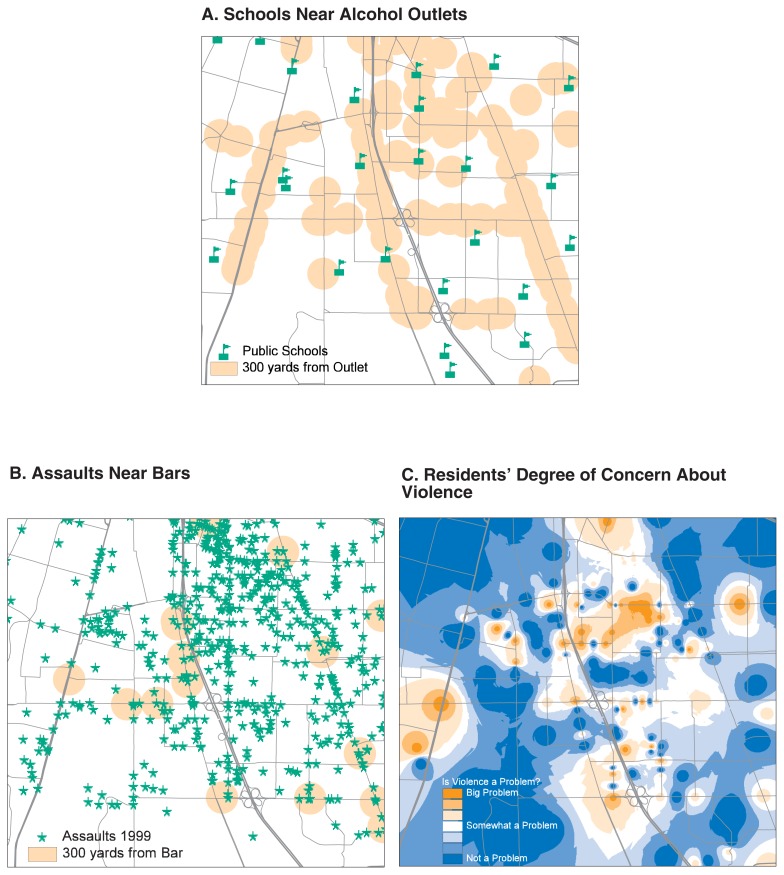
Illustration of the use of geographic information system-based maps to provide descriptive spatial information. Geographic information systems make it possible to organize data so that geographic relationships between objects and events can be measured and quantified.

From a descriptive point of view, the clustering of objects and events and the apparent associations between them as seen on maps may be accidents of geography or urban structure, or be statistical flukes. Geographically, events co-occur in one place because they cannot occur some place else (e.g., traffic crashes do not typically occur in waterways) or because built structures focus events into the same place (e.g., bicycle thefts occur where there are bicycle paths). Statistically, the challenge is to assess the co-occurrence of important events (e.g., alcohol outlets and violence) independent of trivial ones (e.g., their common relationship to retail traffic). To do so, it is necessary to organize the data so that geographic relationships between objects and events can be measured and quantified. The descriptive strengths of GIS are essential to such tasks (Bonham-Carter 1994). Using a GIS, it is possible to investigate, for example, how many schools are within 300 yards of an alcohol outlet (figure 2a), how many reported assaults took place within 300 yards of a bar (figure 2b), and the average degree of concern about violence expressed in neighborhood areas (e.g., using a geographically targeted survey design) (figure 2c). The locations and geographic distributions of these events and survey outcomes can be mapped and their geographic relationships quantified by querying the GIS database. For the neighborhood mapped in figure 2, 6 schools (23 percent of those in the community) are within 300 yards of an alcohol outlet, and 119 assaults (15 percent) occurred within 300 yards of a bar. Spatial analysis can reveal descriptive associations between events and places, providing a preliminary look at potential causal associations between problems and places.

## Spatial Statistics

From a statistical point of view, the patterns of places and events shown on maps can be very misleading, even when quantified. Just as it is possible to find a significant correlation between the height of young people and alcohol use, it is possible to find a significant relationship between locations of gas stations and car crashes. Teenagers are both bigger than younger children and more likely to use alcohol. Gasoline stations and car crashes are both more likely to appear in areas with greater traffic flow. For this reason, it is important to remember that maps and spatial databases only reveal what is queried. If the general characteristics that determine the joint geographic distribution of spatial events are overlooked, or hypotheses about specific relationships are not well constructed, results of statistical analyses can be spurious. This concept is easily forgotten when confronted with a compelling, well-drawn map (Tufte 1983). For example, it is easy to jump to the conclusion that because violent arrests cluster in specific downtown areas, the populations living in those areas are the sources of the violence. It is much more difficult to remember that clusters of events may represent a confluence of forces unrelated to specific local properties of a place (see above).

**Figure 3:**
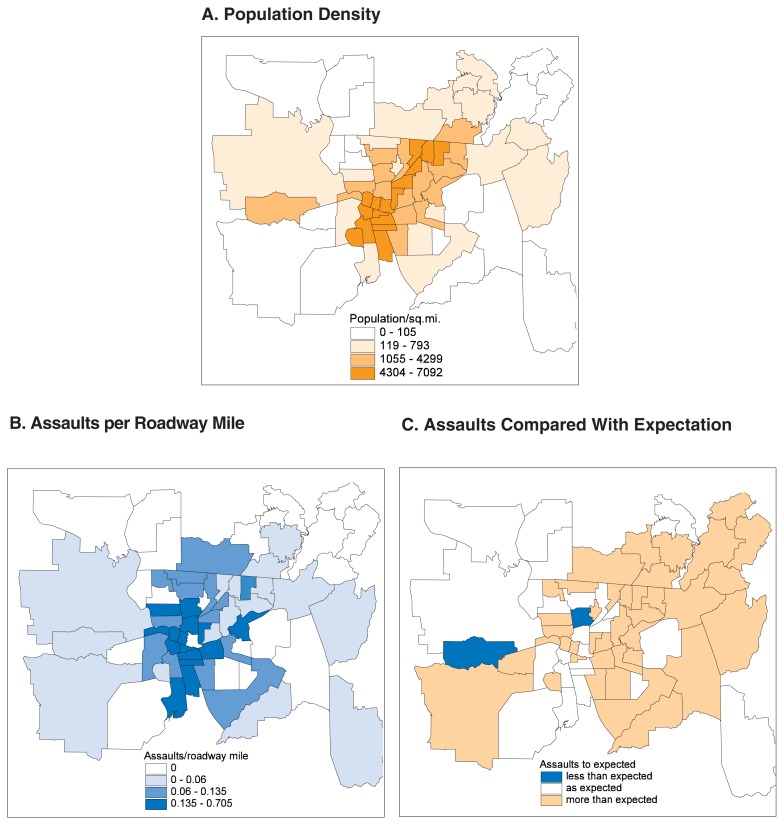
Illustration of the use of geographic information system-based maps with spatial statistics. The geographic distribution of assaults within areas of communities is clearly related to neighborhood populations. Geographic distributions of assaults are, however, also related to characteristics of places and populations living outside of neighborhoods. Taking this spatial information into account, the distribution of hot spots for violence can be better revealed (figure 3c). NOTE: Figure 3c map is based on a model designed to predict assaults based on population density and assaults per roadway mile. “Hot spots,” in orange indicate places where there are more assaults than expected. “Cold spots,” in blue, indicate places where there are fewer assaults than expected.

To avoid the misleading interpretation of geospatial data, researchers have developed geographically based statistical procedures in two subdisciplines called spatial analysis and geostatistics (Bailey and Gattrell 1995). Geostatistics is the subdiscipline of statistical theory that addresses spatial relationships. Spatial analysis is the sub-discipline of statistics that addresses the application of geostatistics to spatial data. Spatial analyses using geostatistical techniques rely on GIS to provide formal mathematical descriptions of spatial relationships (e.g., through matrices representing distances between places) and introduce this information into statistical analyses of geographic (spatial) data. With this information, spatial statistics allow researchers to distinguish the local correlates of problem outcomes (e.g., within neighborhoods) from those correlates related to the larger alcohol environment (e.g., outside of neighborhoods). The results of such analyses can be both quite striking and informative. For example, the geographic distribution of assaults within areas of communities is clearly related to neighborhood populations (see figures 3a and 3b). Geographic distributions of assaults are, however, also related to characteristics of places and populations living outside of neighborhoods (Lipton and Gruenewald in press). Taking this spatial information into account, the distribution of hot spots for violence can be better revealed (figure 3c).

## Geomathematics

To mathematicians, statisticians, ecologists, and many health researchers, space may well be the next frontier. Relationships between objects defined spatially are the subject of purely mathematical endeavors (Okabe et al. 1992), the source of important discourses in mathematical statistics (Cressie 1991), the breakwater for taming a sea of ecological insights in mathematical biology (MacArthur 1972; Levin 1999), and now the concern of alcohol researchers trying to understand and modify community systems that act to support or enhance alcohol-related problems. Some of the alcohol-related problems to which drinkers are prone have a strong social basis (e.g., violence), take place in specific locations (e.g., in and around alcohol outlets), and are related to spatial processes that expose both drinkers and nondrinkers to risk. Harm related to alcohol use occurs in different ways across the geography of communities; alcohol use contributes to traffic crashes and pedestrian injuries, violent assaults, domestic violence, suicide, and other health problems. Thus, mathematical models of the geographic relationships between drinking behavior and problem events will, in the long run, aid communities in the reduction or prevention of alcohol problems. Geomathematics, the mathematical modeling of geographic relationships, will support the development of community models for the reduction of alcohol problems.

Mathematics serves the needs of geographers and ecologists in numerous and important ways: from the projection of maps (e.g., across the different coordinate systems used in cartography) to the efficient construction of system architectures (e.g., the representation of data in GIS), to the spatial algebras of spatial analysis (e.g., specifying distance relationships between objects and events), the mathematics of spatial statistics, and mathematical models of spatial interactions (e.g., representing the forces that shape biodiversity and the distribution of mineral deposits). Sound geomathematical models are essential for transforming statistical studies of the community geography of drinking and problems into significant social policy recommendations.

## Integrating Individual and Environmental Explanations

Although most human behaviors are influenced by a combination of individual and environmental factors, this fact is not often recognized in fields that have been traditionally dominated by survey-based research. This has been true for much of the alcohol research conducted over the past decades. Today, a variety of influences appear to bear upon alcohol problems, and current research suggests a more sophisticated approach. For example, survey research reveals that some people are more likely than others to drive after drinking; maps of communities show that alcohol-related traffic crashes appear to be more frequent in some places than others; and spatial analyses suggest that locations of alcohol outlets influence rates of drinking, drinking and driving, and alcohol-related crashes. Is an explanation of drinking and driving based on the individual sufficient to understand all people who drink and drive? Is an ecological explanation of patterns of traffic crashes sufficient to understand the geographic distribution of alcohol-related crashes? The answer to both questions is “no.” In the former case, the environmental determinants that shape rates of drinking and driving are ignored. In the latter case, individual behavioral predispositions that lead to drinking and driving are neglected.

As this brief sidebar suggests, it would appear that the growth of technologies that enable the mapping of environments in which problem behaviors take place and relating of these measures to individual and aggregate outcomes will lead to explanations that account for both individual and environmental influences in a new manner that goes beyond this overly reductionistic dichotomy. As a simple case in point, just as greater outlet densities may or may not lead to greater rates of violence or alcohol-related crashes at the population level (depending on context) (Lipton and Gruenewald in press; Gruenewald and Treno 2000), individual cases of alcohol-related violence and crashes will be contingent upon a mixture of environmental circumstances and individual predispositions to related behaviors. Mapping the alcohol environment, relating these features of the environment to the spatial distribution of problem events, and analyzing the statistical associations between these measures and drinking behaviors are major goals of current initiatives to study the community geography of alcohol problems. The promise of these areas of research is that they lead to greater understanding of the manifestation of individual problem behaviors in environments that can be regulated and controlled through science-based environmental preventive interventions (Gruenewald et al. in press).
